# Colossal Aggregations of Giant Alien Freshwater Fish as a Potential Biogeochemical Hotspot

**DOI:** 10.1371/journal.pone.0025732

**Published:** 2011-10-05

**Authors:** Stéphanie Boulêtreau, Julien Cucherousset, Sébastien Villéger, Rémi Masson, Frédéric Santoul

**Affiliations:** 1 Université de Toulouse, UPS, INP, UMR 5245 EcoLab (Laboratoire Écologie Fonctionnelle et Environnement), Toulouse, France; 2 CNRS, EcoLab, Toulouse, France; 3 CNRS, UPS, ENFA, UMR 5174 EDB (Laboratoire Évolution et Diversité Biologique), Toulouse, France; 4 Université de Toulouse, UPS, UMR 5174 EDB, Toulouse, France; Biodiversity Insitute of Ontario - University of Guelph, Canada

## Abstract

The ubiquity and fascinating nature of animal aggregations are widely recognised. We report here consistent and previously undocumented occurences of aggregations of a giant alien freshwater fish, the Wels catfish (*Silurus glanis*). Aggregative groups were on average composed of 25 (±10 SD, ranging from 15 to 44) adults with estimated average total biomass of 651 kg (386 – 1132) and biomass density of 23 kg m^−2^ (14 – 40). Aggregations always occurred within the same location. No foraging, reproductive or anti-predator behaviour were observed during the aggregations. A mass-balance model estimated that these colossal aggregations of an alien species can locally release, through excretion only, up to 70 mg P m^−2^ h^−1^ and 400 mg N m^−2^ h^−1^, potentially representing the highest biogeochemical hotspots reported in freshwater ecosystems and another unexpected ecological effect of alien species.

## Introduction

The establishment of vast groups of animals (insect swarms, fish schools, mammal herds or bird flocks) is a phenomenon that has always fascinated humans and scientists. Animal aggregation is ubiquitous, occurring in virtually all taxa, and is driven by a trade-off whereby group members obtain benefits (protection against predators, information to optimize migration route, foraging or mate choice) that are counterbalanced by costs (e.g. intra-specific competition) [1], [2]. One of the most fascinating aspects in the establishment of ephemeral animal aggregations is the occurrence of high local density and biomass. For instance, large numbers of Pacific salmon aggregate in coastal rivers to reproduce, with densities reaching 10 ind. m^−2^ in spawning grounds [3]. Here, we report the consistent occurrence of previously undocumented and colossal aggregations of a giant alien freshwater predator (Wels catfish, *Silurus glanis*). This species is the world's third largest and Europe's largest freshwater fish, originates from Eastern Europe and has been introduced westward [4], [5].

## Results

**Figure 1:**
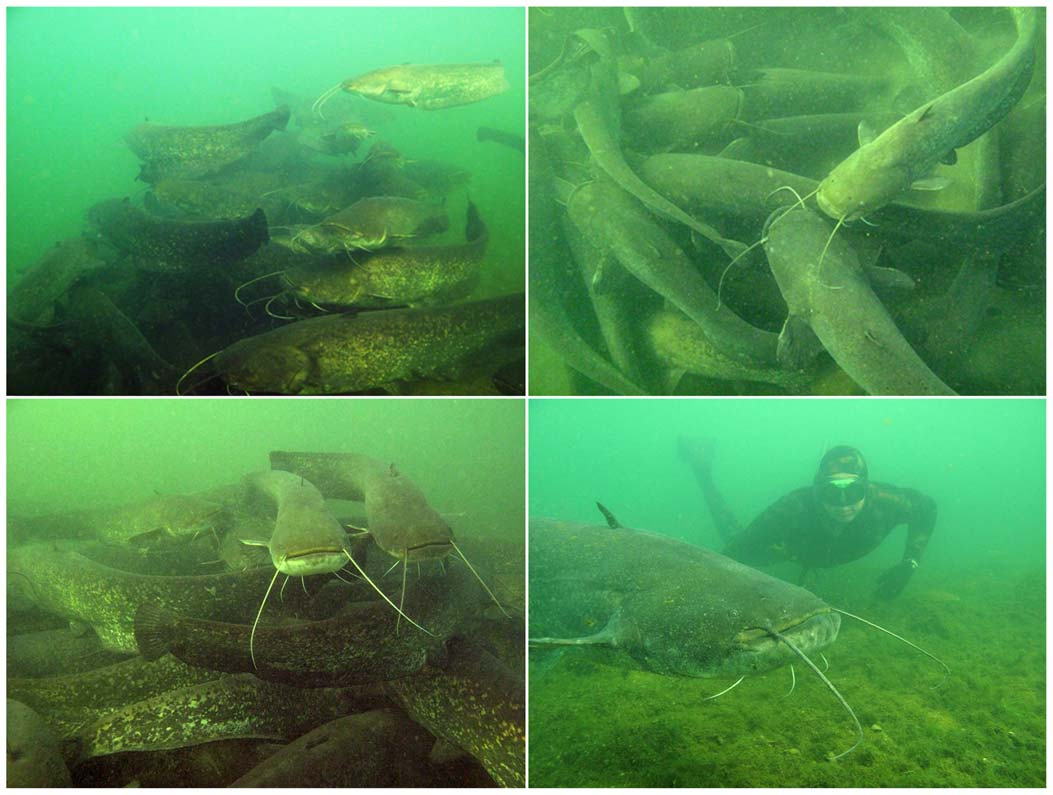
Wels catfish (*Silurus glanis*) aggregations. Bottom-right panel illustrates the size of Wels catfish in the aggregation.

**Figure 2:**
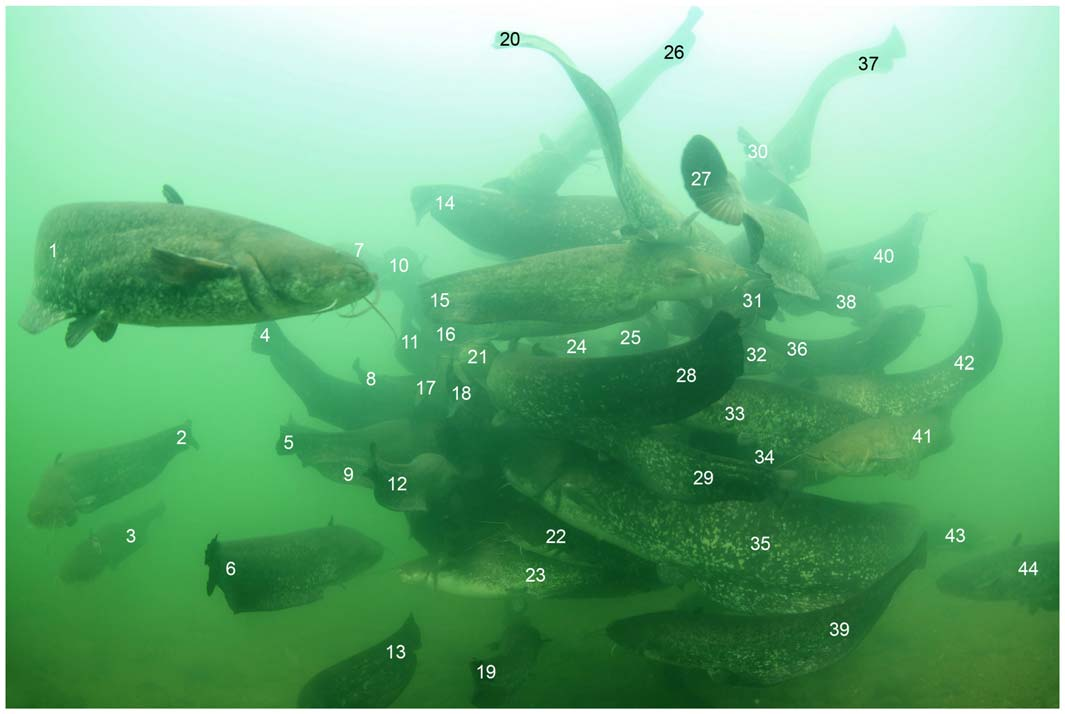
The largest aggregation observed in August 2011. At least fourty two Wels catfish (*Silurus glanis*) can be enumerated.

Monospecific circularly-moving aggregations (Figure 1 and Movie S1) were consistently observed at the same location during 17 snorkelling surveys performed in the Rhône River, France (details in Methods). The number of individuals in the aggregations was estimated to average 25 (±10 SD) adults ranging from 15 to 44 (Figure 2) with estimated body size ranging from 120 to 210 cm and body weight ranging from 12 to 65 kg. Observed aggregations of 15 to 44 individuals represented an estimated total biomass of 386 to 1132 kg, corresponding to an estimated density of 0.5–1.6 ind. m^−2^ and an estimated biomass density of 14–40 kg m^−2^ (Table 1). Using a mass-balance model, we estimated that the colossal aggregations of giant alien catfish locally release, through excretion only, 21–74 mg P m^−2^ h^−1^ and 132–419 mg N m^−2^ h^−1^ (Table 1).

**Table 1:** Mass-balance input variables and estimates.

Mass-balance model inputs	Unit	Average	Min	Max
Density	ind. m^−2^	0.9	0.5	1.6
Total length (TL)	cm	145	120	210
Total biomass (*B_aggregation_*)	kg	651	386	1132
Biomass density	kg m^−2^	23	14	40
**Mass-balance model estimates**				
*Diet: 100% fish*				
P excretion rate	µmol P m^−2^ h^−1^	1366	810	2376
P excretion rate	mg P m^−2^ h^−1^	42	25	74
N excretion rate	µmol N m^−2^ h^−1^	17006	10201	29924
N excretion rate	mg N m^−2^ h^−1^	241	143	419
*Diet: 80% fish & 20% crayfish*				
P excretion rate	µmol P m^−2^ h^−1^	1167	692	2029
P excretion rate	mg P m^−2^ h^−1^	36	21	63
N excretion rate	µmol N m^−2^ h^−1^	15847	9396	27560
N excretion rate	mg N m^−2^ h^−1^	222	132	386

Density, total length, biomass and excretion rates were calculated for average (n = 25 individuals), minimal (n = 15 individuals) and maximal (n = 44 individuals) aggregation sizes. Excretion rates were also calculated for two diet compositions.

## Discussion

Evidence suggests that the mechanism responsible for the observed aggregations were not associated with schooling behavior, reproduction, foraging or anti-predator behavior. Indeed, individuals were active, always swimming, but were not all pointing in the same direction as in polarized shoals. No synchronous movements were observed and, contrary to schooling fish that maintain a minimum distance between conspecifics, individuals were swimming while rubbing against each other. Therefore, the observed aggregations do not represent fish schools *sensu stricto* [6]. It is unlikely that they were linked to reproduction since no mating behaviours were observed and groups occurred throughout the year at temperatures below the spawning threshold [5]. In addition, foraging behaviour was unlikely since no prey was captured and no foraging behaviour was displayed. Additionally, all individuals were large enough to be released from any predation risk.

Because of the very large size of these alien individuals (approximately five times heavier than native fish species), their aggregation can potentially lead to important functional consequences in recipient ecosystems. For instance, defecation and excretion from dense aggregations of fish that rest over coral reefs can provide important quantity of nitrogen and phosphorus that subsequently increase the growth rate of corals [7]. In some cases fish can translocate nutrients within the ecosystem by feeding in one location while defecating in another [7], [8]. Additionnally, heterogenous spatial distribution of fish can also create biogeochemical hotspots, i.e. places where nutrient release by animals exceeds the need of primary producers [9].

Here, the observed aggregations of alien fish potentially represent the highest biogeochemical hotspots ever reported for freshwater ecosystems, as our estimates correspond to 83–286 fold and 17–56 fold the maximal fish excretion values for P and N, respectively, reported in the literature (e.g. [9], [10]). Therefore, these aggregations can potentially have strong implications on ecosystem functioning since these fish may translocate nutrients from their feeding areas, concentrate locally these nutrients in the aggregation area and subsequently affect primary production and nutrient cycling. Therefore, this phenomenon represents another example of unexpected potential ecological impacts of alien species (e.g. [11]).

## Methods

### Observations

Wels catfish (*Silurus glanis*) were observed by snorkelling in a stretch (mean length = 400 m, mean width = 200 m) of the Rhône River located downstream of Lyon (France). The studied stretch has a mean discharge of 150 m^3^ s^−1^, ranging annually from 50 to 200 m^3^ s^−1^. Seventeen surveys (2-hour long) were performed in the early afternoon by the same person (RM) at the same place, when permitted by water visibility. At this place, current is relatively slow, with a mean depth of 5 m and a substrate composed of gravels and pebbles. The surveys were performed from May 2009 to August 2011 on the following dates: May (05/19/2009 & 05/20/2009); June (06/12/2009); March (03/24/2010); April (04/28/2010); September (09/05/2010); October (10/09/2010, 10/18/2010, 10/26/2010 & 10/31/2010); December (12/03/2010); February (02/08/2011 & 02/11/2011); June (06/30/2011); July (07/06/2011) and August (08/21/2011 & 08/24/2011). On some occasions, aggregations were pictured or filmed (n = 8) from a distance of less 2 m from fishes, without resulting in any perceptible disturbance.

### Biomass and density estimates

Estimated aggregations size averaged 25 adults Wels catfish and ranged from 15 to 44 adults among the 17 surveys. The groups were mainly composed of 120-cm long individuals, followed by some 210-cm long individuals and few 170-cm long individuals (a 60-cm long individual, observed at a few occasions, was not used for the subsequent estimation of aggregation biomass, density and excretion). Estimated body-length distribution was computed as followed: 66% 120 cm TL, 14% 170 cm TL and 20% 210 cm TL. Total biomass of the aggregation was estimated assuming a sex ratio of 1∶1 and using the length-weight relationships for females and males Wels catfish (Table 2). Total biomass density of the aggregation was estimated assuming that aggregations had a circular-like shape with a maximal estimated diameter of 6 m.

**Table 2:** Mass-balance model parameters.

Mass-balance model parameters	Unit	Value or relation	References
Fish P concentration ([*P*]_fish_)	% of wet mass	0.5	[17], [18]
Crayfish P concentration ([*P*]_crayfish_)	% of wet mass	0.16	[13], [18]
Fish N concentration ([*N*]_fish_)	% of wet mass	2.54	[17], [18]
Crayfish N concentration ([*N*]_crayfish_)	% of wet mass	1.6	[13], [18]
Daily ration (*DR*)	% of wet mass day^−1^	1.32	[5]
Specific growth rate (*SGR*)	% day^−1^	0.066*	[19]
Assimilation efficiency of P (*AE_P_*)	%	72	[14]
Assimilation efficiency of N (*AE_N_*)	%	80	[20]
Female weight (*W_female_*)	g	0.0038 TL^3.1295^	[16]
Male weight (*W_male_*)	g	0.0104 TL^2.9133^	[16]

*The specific growth rate is the mean of specific growth rates of the biggest individuals.

### Excretion estimates

Nitrogen and phosphorus excretion rates were calculated from a mass-balance model [12] commonly used in fish bioenergetics modelling [13] as follows:
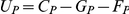



where *U_P_* is the mass of P excreted, i.e., lost in urine (g), *C_P_* is the mass of P consumed (g), *G_P_* is the mass of P allocated to growth (g) and *F_P_* is the mass of P lost in faeces (g).

Excreted P (or N) was estimated as the difference between the P (N) consumed and the P (N) lost in faeces and allocated to growth. Faecal loss can be accounted for as a direct proportion of consumption [14] by applying a gross assimilation efficiency *AE_P_* (*AE_N_*) for a given prey type, simplifying equation 1 to:
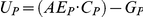



The mass of P (N) consumed was calculated as the product of the mass of prey consumed and the concentration of P (N) in prey tissue (*[P]_prey_* and *[N]_prey_*). The mass of prey was deduced from the average daily ration (*DR*) of Wels catfish of size ranges 51–70 cm, 71–100 cm and 40–160 cm from June, July, August and September (reviewed in [15]). Two diet compositions were simulated, i.e. 100% fish or 80% fish and 20% crayfish [15], [16].

The mass of P allocated to growth was calculated as the product of the growth rate and the concentration of P (N) in predator tissue (*[P]_pred_* and *[N]_pred_*). The growth rate was estimated from the product of the specific growth rate (*SGR*). The model becomes:
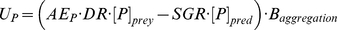


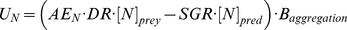



The list of parameter values is detailed in Table 1.

## Supporting Information

Movie S1 Movie showing the aggregations of Wels catfish (*Silurus glanis*).
(WMV)

## References

[1] Krause J, Ruxton GD (2002). Living in groups.. Oxford, UK: Oxford University Press.

[2] Parrish JK, Edelstein-Keshet L (1999). Complexity, Pattern, and Evolutionary trade-Offs in Animal Aggregation.. Science.

[3] Schindler DE, Scheuerell MD, Moore JW, Gende SM, Francis TB (2003). Pacific salmon and the ecology of coastal ecosystems.. Front Ecol Environ.

[4] Stone R (2007). The last of the Leviathans.. Science.

[5] Copp GH, Britton JR, Cucherousset J, García-Berthou E, Kirk R (2009). Voracious invader or benign feline? A review of the environmental biology of European catfish *Silurus glanis* in its native and introduces ranges.. Fish Fish.

[6] Pitcher TJ (1983). Heuristic definitions of fish shoaling behaviour.. Animal Behav.

[7] Meyer JL, Schultz ET, Helfman S (1983). Fish Shools: An Asset to Corals.. Science.

[8] Krone R, Bshary R, Paster M, Eisinger M, van Treeck P (2008). Defecation behaviour of the Lined Bristletooth Surgeonfish Ctenochaetus striatus (Acanthuridae).. Coral Reefs.

[9] McIntyre PB, Flecker AS, Vanni MJ, Hood JM, Taylor BW (2008). Fish distributions and nutrient cycling in streams: can fish create biogeochemical hotspots?. Ecology.

[10] Schaus MH, Vanni MJ, Wissing TE, Bremingan MT, Garvey JE (1997). Nitrogen and phosphorus excretion by detritivorous gizzard shad in a reservoir ecosystem.. Limnol Oceanogr.

[11] Cucherousset J, Olden JD (2011). Ecological impacts of non-native freshwater fishes.. Fisheries.

[12] Nakashima BS, Leggett WC (1980). The role of fishes in the regulation of phosphorus availability in lakes.. Can J Fish Aquat Sci.

[13] Hanson PC, Johnson TB, Schindler DE, Kitchell JF (1997). Fish Bioenergetics 3.0.. Sea Grant Technical Report, University of Wisconsin Sea Grant Institute, Madison, USA.

[14] Nakashima BS, Leggett WC (1980). Natural sources and requirements of phosphorus for fishes.. Can J Fish Aquat Sci.

[15] Syväranta j, Cucherousset J, Kopp D, Crivelli A, Céréghino R (2010). Dietary breadth and trophic position of introduced European catfish *Silurus glanis* in the River Tarn (Garonne River basin), Southwest France.. Aquat Biol.

[16] Alp A, Kara C, Üçkardeş F, Carol J, García-Berthou E (2010). Age and growth of the European catfish (*Silurus glanis*) in a Turkish Reservoir and comparison with introduced populations.. Rev Fish Biol Fisheries.

[17] Penszak T (1985). Phosphorus, nitrogen, and carbon cycling by fish populations in two small lowland rivers in Poland.. Hydrobiologia.

[18] Davis JA, Boyd CE (1975). Concentrations of selected elements and ash in bluegill (*Lepomis macrochirus*) and certain other freshwater fish.. Trans Am Fish Soc.

[19] Britton JR, Pegg J, Sedwick R, Page R (2007). Investigating the catch returns and growth rate of Wels catfish, *Silurus glanis*, using mark-recapture.. Fisheries Management and Ecology.

[20] Brett JR, Groves TDD (1979). Fish Physiology, Vol..

